# Random Lasing Detection of Mutant Huntingtin Expression in Cells

**DOI:** 10.3390/s21113825

**Published:** 2021-05-31

**Authors:** Sergio de Armas-Rillo, Felipe Fumagallo-Reading, Diego Luis-Ravelo, Beatriz Abdul-Jalbar, Tomás González-Hernández, Fernando Lahoz

**Affiliations:** 1 Departamento de Física, Instituto Universitario de Estudios Avanzados en Física Atómica, Molecular y Fotónica (IUdEA), Universidad de La Laguna, 38206 Santa Cruz de Tenerife, Spain; flahoz@ull.es; 2 Departamento de Ciencias Médicas Básicas, Instituto de Tecnologías Biomédicas (ITB), Universidad de La Laguna, 38320 Santa Cruz de Tenerife, Spain; ffumagal@ull.es (F.F.-R.); dluisrav@ull.edu.es (D.L.-R.); tgonhern@ull.edu.es (T.G.-H.); 3 Departamento de Matemáticas, Estadística e Investigación Operativa, Universidad de La Laguna, 38206 Santa Cruz de Tenerife, Spain; babdul@ull.edu.es

**Keywords:** random lasing, Huntington’s disease, PCA, LDA, optical sensor, optical diagnostics

## Abstract

Huntington’s disease (HD) is an autosomal dominant, incurable neurodegenerative disease caused by mutation in the huntingtin gene (*HTT*). *HTT* mutation leads to protein misfolding and aggregation, which affect cells’ functions and structural features. Because these changes might modify the scattering strength of affected cells, we propose that random lasing (RL) is an appropriate technique for detecting cells that express mutated *HTT*. To explore this hypothesis, we used a cell model of HD based on the expression of two different forms—pathogenic and non-pathogenic—of *HTT*. The RL signals from both cell profiles were compared. A multivariate statistical analysis of the RL signals based on the principal component analysis (PCA) and linear discriminant analysis (LDA) techniques revealed substantial differences between cells that expressed the pathogenic and the non-pathogenic forms of *HTT*.

## 1. Introduction

Although it was first described more than 50 years ago [1,2], investigations of potential applications of the optical phenomenon known as random lasing (RL) have seen tremendous growth in recent decades. These investigations initially focused on inorganic systems [3,4,5,6,7], but biological systems were soon included in the research of RL. In short, for RL to appear, two fundamental ingredients are needed: light propagating through an amplifying medium (such as a laser-dye-doped medium) and scattering centers throughout that medium. Inside mediums with sufficient scattering strength, the propagating light may be scattered in a way in which it eventually returns to a previous scattering center, forming a closed optical loop. Under appropriate conditions, these closed loops become laser oscillators and, eventually, laser emission occurs [4,8]. Indeed, biological environments show an intrinsic optical scattering nature (as they tend to have a high scattering strength). Consequently, they have proved to be excellent media for RL action [9]. Many biological systems have been studied in recent years: the bovine heart [10], pig tissue (fat, nerve, muscle and skin) [11], insect wings [9], and rat and mouse uterus [12], as well as different human tissues, such as bone [13,14], brain [15], colon, kidney, and breast [16,17]. RL has also been obtained from cells in suspension in a cytometer, with subcellular properties modifying the emission of the laser [18]. Interestingly, differences in the scattering properties of tissues can have an effect on the RL characteristics of the gain medium. For example, RL has been successfully used to distinguish between healthy and cancerous cells and tissues [16,17,18]. However, so far, RL has not been reported in biological systems with respect to neurodegenerative pathologies.

Huntington’s disease (HD) is an autosomal dominant, chronic, incurable neurodegenerative disease caused by an anomalous expansion of the trinucleotide sequence CAG, which encodes the amino acid glutamine within the Huntingtin gene (*HTT*; [19]). The mutation results in longer stretches of polyglutamine (poly-Q) tracts, which are prone to misfolding and accumulation in the form of cytosolic and intranuclear aggregates [20,21]. Monomeric and oligomeric fragments resulting from the proteolytic cleavage of mutant *Huntingtin* (mHTT) interfere with critical cellular functions, leading to excitotoxicity, synaptic dysfunction, mitochondrial failure, and cell death [22]. HD can manifest itself when the number of CAG repeats is greater than 36, with reduced penetrance between 36 and 39 repeats and high penetrance when exceeding 39 repeats [23]. HD is characterized by motor, cognitive, and psychiatric symptoms that appear around 40 years of age and progress to death in 10–20 years [24,25]. It should be noted that although neurological symptoms become evident in midlife, the degenerative process is initiated a few decades before [19,24,26,27]. Thus, there is interest in finding neuroprotective treatments that can be introduced in pre-manifestation stages and in biomarkers that detect mHTT carriers who are at risk of suffering from HD. Clinical trials have shown promising results with therapeutic approaches based on gene therapy [22,28], but the identification of biomarkers that allow us to predict the fate of mHTT carriers remains a challenging task. Efforts directed to both image and biofluid tests have revealed some candidates, but they still need extensive evaluation before their translation into the clinical practice [29,30,31]. In this context, we hypothesized that the morphological/structural changes induced by mHTT expression can modify the scattering strength and the optical behavior of an environment when light propagates through it, resulting in differences in the RL signal compared to the signals arising from cells that express the wild-type *HTT* form.

To assess this hypothesis, we used a cell model of HD based on the stable transfection of the pathogenic (HTT-Q74) vs. the non-pathogenic (HTT-Q23) expansion of *HTT* in a neuronal cell line. RL emissions were obtained from transfected (EGFP-HTT-Q23 or EGFP-HTT-Q74) and un-transfected cells (non-transfected or NT), resulting in a high-dimensional spectral dataset. This RL dataset was analyzed by using a multivariate statistical analysis based on two techniques: principal component analysis (PCA) and linear discriminant analysis (LDA).

## 2. Materials and Methods

### 2.1. Cell Transfection

**Figure 1:**
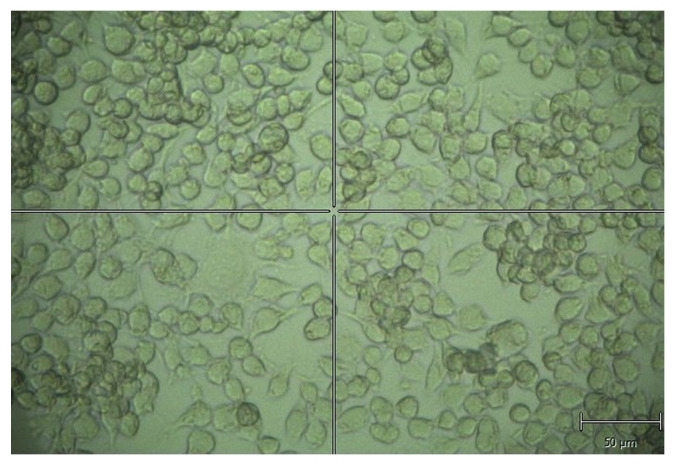
Example of the studied cell culture.

The mouse neuroblastoma cell line Neuro-2a (N2A; ATCC^®^ CCL-131) was cultured in Dulbecco’s Modified Eagle’s Medium (DMEM; Biowest, L104) supplemented with fetal bovine serum (FBS, 10%) and 1% penicillin–streptomycin. Cultures were maintained in a humidified incubator at 37 ºC and 5% CO_2_. Semiconfluent cultures were transfected with pEGFP-Q23 or pEGFP-Q74 (Plasmids #40261 and #40262, Addgene), which contained normal or expanded poly-Q tracts within *HTT* exon 1 in frame with EGFP, respectively. Lipofectamine 2000 reagent (Thermo Fisher Scientific) was used at a ratio of 2:1 according to the manufacturer’s protocol. After 10–15 d of selection in G418 (1 mg/mL, Sigma-Aldrich, A1780), individual clones were expanded in multi-well plates and examined for expression by using a Western blot and immunofluorescence for GFP. Positive clones were identified and used for subsequent experiments. When cellular confluence was nearly 100% (see Figure 1), cultures were fixed in 4% paraformaldehyde in 0.1 mM phosphate buffer saline (137 mM NaCl, 2.7 mM KCl, 10 mM Na_2_HPO_4_, 1.8 mM KH_2_PO_4_, pH 7.4; PBS) for 20 min, and were then washed in PBS three times.

HTT-Q23 and HTT-Q74 expression was confirmed with a Western blot for GFP. Cell cultures were washed with phosphate buffered saline and harvested in ice-cold PBS. Cell suspensions were centrifuged for 5 min at 1000× *g* (4 °C), and the pellets were resuspended in 40–50 μL M-PER (Thermo Fisher Scientific) containing protease inhibitor cocktail according to the manufacturer’s instructions (Sigma-Aldrich). Lysates were centrifuged at 17,000× *g* for 5 min, and the supernatants were collected. Proteins were quantified using the bicinchoninic acid method and with bovine serum albumin as standard. Fifteen micrograms of protein were diluted in Laemmli’s loading buffer (62.5 mM Tris-HCl, 20%, 2% sodium dodecyl sulfate (SDS), 1.7% β-mercaptoethanol, and 0.05% bromophenol blue, pH 6.8), heated (90 °C, 5 min), resolved by 12% SDS-polyacrylamide gel, and transferred to nitrocellulose membranes. Blots were blocked for 1 h at room temperature with 5% nonfat dry milk in TBST (250 mM NaCl, 50 mM Tris, pH 7.4, and 0.05% Tween20) and incubated overnight at 4 °C in blocking solution with mouse monoclonal anti-GFP (1:2000; Roche) and anti-TUBA4A/α-Tubulin (1:30,000; Sigma-Aldrich) antibodies. After several rinses in TBST, the membranes were incubated for 1 h in horseradish peroxidase-conjugated anti-mouse-IgG (1:20,000; Jackson-ImmunoResearch Laboratories) and rinsed. Immunoreactive bands were detected using an enhanced chemiluminiscence substrate (Immun-Star; Bio-Rad) and a Chemi-Doc gel documentation system (Bio-Rad).

### 2.2. Cell Culture Staining

**Figure 2:**
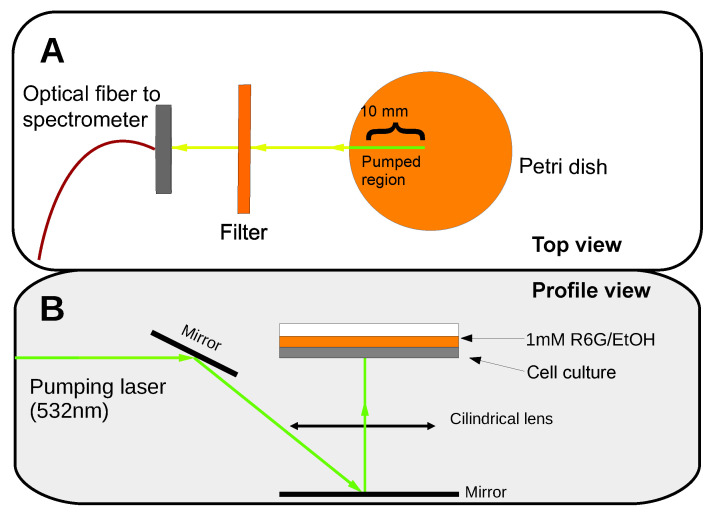
Experimental setup. (**A**) Top view of the experiment with the pumped region marked in green. (**B**) Profile view of the setup.

PBS was substituted with 400 μL of a dying dilution of 1 mM Rhodamine 6G in ethanol (R6G/EtOH). The dye solution’s volume was just enough to entirely cover the cells. Then, the stained cellular cultures were placed in the experimental setup illustrated in Figure 2.

### 2.3. RL Measurements

A frequency-doubled Nd-YAG pulsed laser (Continuum, Surelite S I-20) emitting pulses at 532 nm was used as the pump source. The laser pulses had a frequency of 20 Hz, and the temporal pulse width was about 8 ns. Two linear polarizers (Thorlabs SM1PM5) were used to modulate the intensity of the pump beam. The pump laser power was measured using a pyroelectric energy sensor. An adjustable slit was placed in the optical path to select the most apparently homogeneous laser beam area with a length of 10 mm. A cylindrical lens was used to focus the pump beam on the sample with normal incidence to form a horizontal line that was 10 mm long and 0.3 mm wide. The pump’s energy density distribution on the excitation area had a “top-hat” like profile in the long direction—with some heterogeneities along the 10 mm length—and a Gaussian profile along the perpendicular axis. A system of mirrors redirected the beam so that it illuminated the sample from below. The signal was collected along the stripe pump direction using an optical fiber with a core diameter of 200 μm (Thorlabs M25L05) connected to a spectrometer (ANDOR SR-303I-B) coupled to a CCD (Newton 970EMCCD) detector with a resolution of 0.04 nm (see Figure 2). We would like to remark that, from the experimental point of view, to optimize the RL signal, it was critical to pump the Petri dish samples from below. The interaction of the light emitted from R6G with the cells was higher under this configuration rather than when pumping from above. Moreover, if the samples were optically pumped from above, an intense amplified spontaneous emission (ASE) from the R6G solution layer, which stood over the cell culture, overlapped with the RL emission. However, the RL signal was notably increased when the optical pumping was performed from below the sample and the ASE from the liquid phase of R6G was hindered.

### 2.4. Statistical Analysis

First, PCA was performed to reduce the spectral variables to a few principal components (PCs) while retaining the maximum variation in the data. The number of PCs selected was determined with the Kaiser criterion [32], which states that components should be retained if their eigenvalues are greater than or equal to one. Then, the PC scores were used as the input for the LDA to attempt to discriminate among the three types of cell cultures (NT, HTT-Q23, and HTT-Q74). In order to validate the PCA–LDA model, the dataset was randomly split into a training set—consisting of 70% of the spectral data—and a validation set with the other 30% of the data. The performance of the model was first evaluated by applying leave-one-out cross-validation in the training set. The model was also validated by classifying the samples in the validation set. All statistical analyses were performed using IBM^®^ SPSS Statistics 25.0 software package (IBM Corp., New York, NY, USA) for Windows.

## 3. Results and Discussion

**Figure 3:**
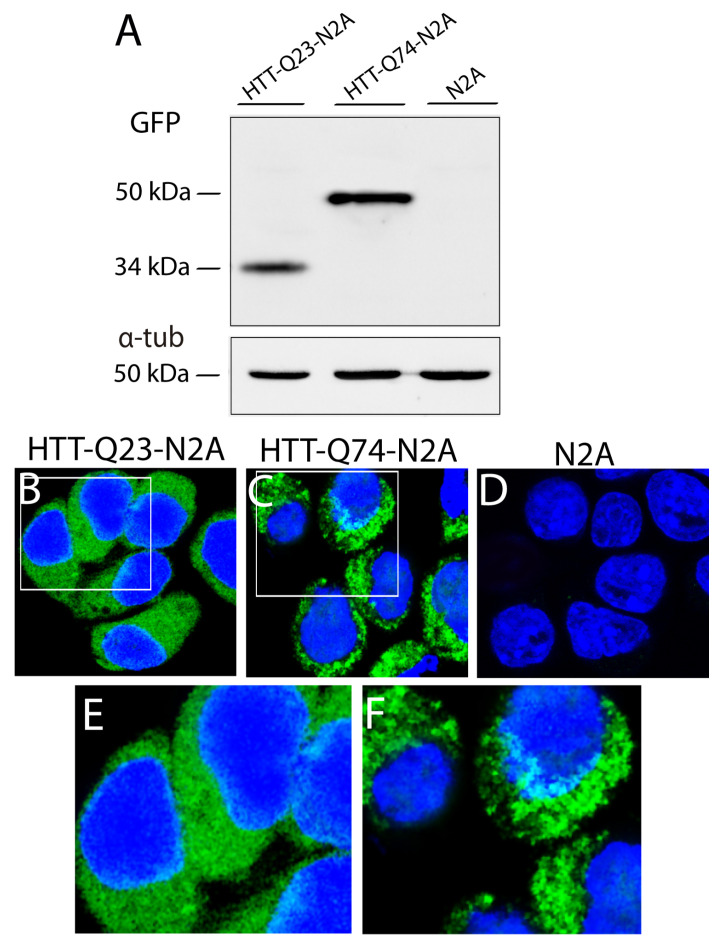
Western blot for GFP (**A**) and fluorescence microscopy (**B**–**D**) in EGFP-HTT-Q23- (HTT-Q23-N2A), EGFP-HTT-Q74- (HTT-Q74-N2A), and non-transfected N2A cells. (**E**,**F**) Insets in (**B**,**C**). The immunoreactive GFP band in EGFP-HTT-Q23-expressing cells was at 34 kDa, and in EGFP-HTT-Q74-expressing cells, it was at 50 kDa. No bands were detected in the NT cells. We can see that while HTT-Q23 expression showed homogenous cytosolic labeling (**B**–**E**), HTT-Q74 expression showed a granular labeling pattern (**C**–**F**). No green fluorescent labeling was detected in the NT cells (**D**). Cell nuclei were stained with 2-[4-(Aminoiminomethyl)phenyl]-1H-Indole-6-carboximidamide hydrochloride (DAPI). Bar in **D** (for **B**–**D**): 10 μm.

The expression of the Q23 and Q74 expansions in N2A cells was assessed with a Western blot for GFP. As shown in Figure 3A, HTT-Q23 was detected as a single band at approximately 34 kDa (Figure 3A, lane 1), and HTT-Q74 was detected as a single band at 50 kDa (lane 2). In addition, confocal microscopy revealed differences in the labeling patterns of both poly-Q expansions. While HTT-Q23 showed a diffuse and homogeneous distribution throughout the cytosol (Figure 3B,E), HTT-Q74 displayed a granular labeling, suggesting that it formed cytosolic aggregates (Figure 3C,E). No intranuclear inclusions were detected.

**Figure 4:**
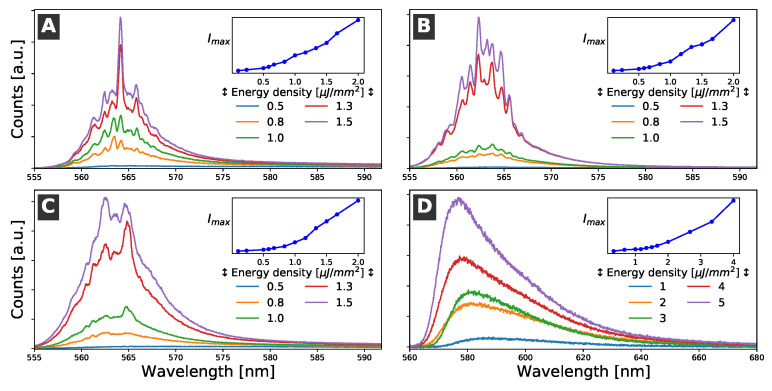
Evolution of the emission of the sample with increases in the pumping power density. The blueshift of the spectra, the emergence of narrow (FWHM < 1 nm) lines, and the dramatic increase in emission intensity were all due to the coherent RL. (**A**) NT (non-transfected N2A cells). (**B**) HTT-Q23 (N2A cells transfected with the non-pathogenic pEGFP-Q23). (**C**) HTT-Q74 (N2A transfected with the pathogenic pEGFP-Q74). (**D**) Petri dish without cells. In this case, an ASE, but not RL, was detected.

The emission spectra of the samples were monitored as a function of the pumping energy density. At relatively low pumping energy density, the spectra showed the characteristic emission band of R6G in ethanol, which was formed by a broad visible band with a maximum around 587 nm and a full width at half maximum (FWHM) at about 50 nm. The emission of the R6G solution was isotropic and increased linearly with the pumping energy density. However, when the pump power reached a certain value, the features of the emission spectrum dramatically changed. The emission intensity suddenly increased and a set of narrow emission lines appeared. The FWHM of the narrowest emission lines was less than 1 nm. Moreover, at high pump power values, the irradiated volume of the R6G solution acted as a waveguide, and the emission light could be easily detected by placing a optical detection fiber in the direction of the pump stripe (see Figure 2) [33]. The changes in the emission spectra and emission intensities are shown in Figure 4. Similar results were obtained for all of the samples.

The narrow emission lines observed with the high pump power were attributed to coherent RL modes formed by the interaction of the optical-gain liquid medium with the cells, which acted as scattering centers. Studies of the dependence of the emission spectra of R6G solutions were also conducted on dishes without cells. In these cases, no evidence of RL was observed; only an ASE of the dye solution was detected, and it had an FWHM of about 12 nm, which was much larger than the narrow lines (about 1 nm) ascribed to the RL modes.

The RL pump energy density thresholds detected for the three cellular types were around 0.5–0.7 μJ/mm^2^. This was a relatively low pump density, and no evidence of photobleaching was observed even at pump densities that were five times larger than the threshold. Changes in the RL threshold in healthy and cancerous tissues have been previously observed and considered as criteria for cancer diagnostic purposes [17]. However, in our case, the RL thresholds were similar for all of the samples and could not be determined with better accuracy, which was probably because of small fluctuations in the cell confluence. Therefore, it was experimentally difficult to distinguish the different cell cultures according to the measurement of the RL threshold or by using changes in the RL emission intensity. Moreover, other characteristic features of the RL emission, such as emission intensity, FWHM of the RL emission modes, or number of narrow RL lines, were similar in all of the samples; consequently, they could not be satisfactorily used to distinguish among them.

In a recent paper, Huefner et al. [34] successfully conducted Raman spectroscopy for the diagnosis of HD by observing differences in the analysis of the Raman signals of blood serum in patients with or without the disease. They applied a multivariate statistical analysis with PCA–LDA to examine their spectroscopic data. Therefore, we decided to follow a similar strategy in order to analyze our high-dimensional RL dataset. First, we applied PCA to reduce the dimensionality of the spectral data, and then LDA was performed to elucidate if RL could distinguish HD-induced cell cultures. For this purpose, 1000 single-shot measurements were performed on each cell type. They were obtained by recording 100 spectra from 10 different pumped regions, which were obtained by just rotating the petri dishes. The pump energy density was set to about 3 μJ/mm^2^, which was well above RL threshold and for which no photobleaching of the emission was observed; it was also well below the recommended limit of exposure due to health risks with this type of laser irradiation—around 200 μJ/mm^2^ [35]. We randomly selected 70% of the spectra to build the statistical model and left the other 30% to validate the model.

Most of the information of the RL spectra was contained in the spectral range between 555 and 575 nm (see Figure 4). Therefore, PCA was conducted in this spectral range. This analysis showed that 28 PCs had eigenvalues greater than 1 and were able to explain about 92% of the variability in the data. In particular, the first two components (PC1 and PC2) accounted for over 67% of the variability. Interestingly, some studies have suggested that PCA per se may be enough to distinguish between different biological samples [34,36].

**Figure 5:**
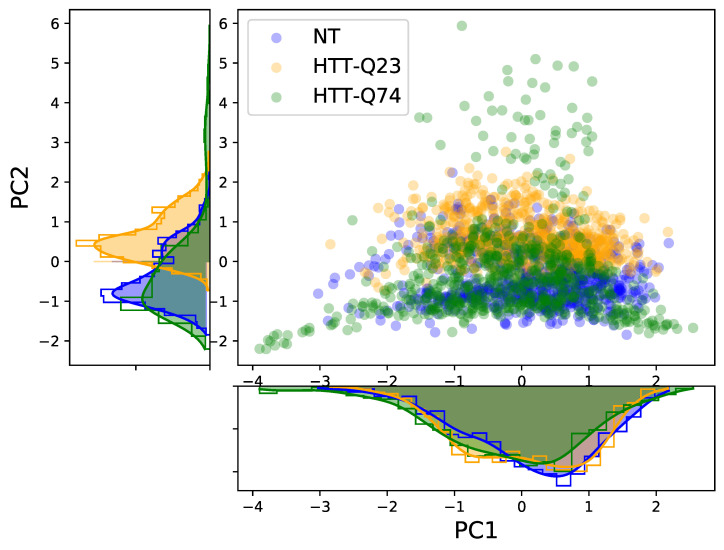
Scores of the two main PCs. Their respective distributions are also represented.

Figure 5 shows the scatter plot of the PC1 and PC2 scores for the three cellular samples. We can see that the PC1 scores showed a strong overlap across all cell types, and only the PC2 scores of HTT-Q23 were shifted from those of NT and HTT-Q74. In addition, we examined the 3D score plot of the first three PCs, but, again, it was not possible to clearly discriminate among the groups. These results suggest that, in our case, PCA was not able to visually distinguish the three different cellular types. Therefore, LDA was additionally conducted using the 28 PCs extracted by the PCA as input. Because the goal was to discriminate among three different cellular profiles (NT, HTT-Q23, and HTT-Q74), a maximum of two discriminant functions (LD1 and LD2) could be computed. LDA revealed that both discriminant functions were significant, with LD1 and LD2 accounting for 64.4% and 35.6% of the variation between groups, respectively.

**Figure 6:**
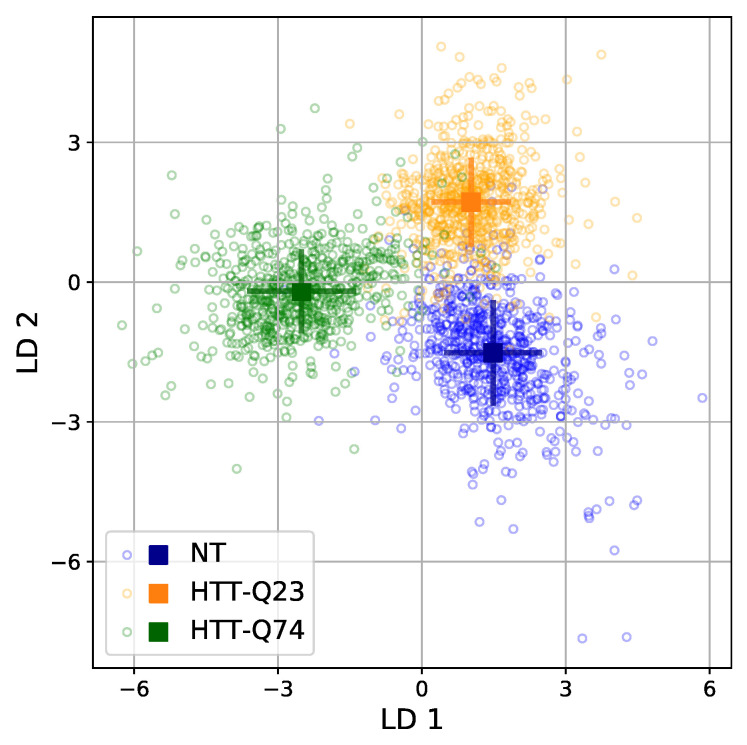
LDA scores for the RL spectra in the training set. Colored squares show the positions of the centroids of each group; the corresponding lines illustrate the standard deviation of the values inside each group.

Figure 6 shows a scatter plot of the scores of LD1 and LD2 for each spectrum in the training set. The centroids of the distributions are indicated with a square, and the error bars represent the standard deviation of the distributions. It can be observed that LD1 and LD2 clearly discriminated among the three cellular groups. The first discriminant function mainly separated HTT-Q74 from HTT-Q23 and NT, while the second discriminated between HTT-Q23 and NT. Concretely, the HTT-Q74 cell samples were located on the negative part of LD1, the HTT-Q23 cell samples were located on the positive part of LD1 and the positive part of LD2, and the NT samples were located on the positive part of LD1 and the negative part of LD2. In addition, Table 1 shows the centroids of the groups (the mean values of both functions for the spectra of the same cell profile).

We built a model based on PCA and LDA that appeared to be able to discriminate between cells that expressed the non-pathogenic or pathogenic form of Huntingtin. This model was obtained by using a randomly selected training set consisting of 70% of the 3000 RL spectra that made up the original dataset. In order to validate it, we first used leave-one-out cross-validation in which each sample was left out—one at a time—from the training dataset; then, the model was rebuilt, and the removed case was classified in this new model. Finally, we also evaluated the performance of the model by classifying the remaining 30% of the spectra in the validation set. In Table 2, we can see the results of both validations.

The discriminant power of our model was apparent with the results shown in Figure 6. The model achieved an overall classification accuracy of 94.4% in the training set. As can be seen, the highest success rate was obtained for the HTT-Q23 group, for which the model was able to correctly classify 95.4% of the spectra. The success ratios for the other two cell profiles were also very high: 93.9% for NT and 94.0% for HTT-Q74. Regarding the leave-one-out cross-validation, the results were very similar. The model achieved an overall classification accuracy of 94.4%, with the highest percentage for HTT-Q23 (95%) and a slightly lower success rate for NT (93.7%) and HTT-Q74 (93.6%). Finally, the model was also able to correctly classify most of the samples in the validation set, reaching an overall accuracy of 94.2%. From the above results, we can confidently say that our model has a strong discriminant power. Moreover, the same transfection vector, procedure, and reporter were used for the GFP-Q23-N2A and GFP-Q74-N2A cell lines, which showed similar expression levels. Therefore, the results of the analysis of RL discrimination between those cell types seem to be due to physicochemical differences between the cells rather than their mHTT expression levels.

## 4. Conclusions

We successfully used random lasing to discriminate among three cell profiles: non-transfected N2A cells, which were used as a control (NT), N2A cells transfected with a non-pathogenic form of Huntingtin (HTT-Q23), and N2A cells transfected with a pathogenic form of Huntingtin (HTT-Q74), which was used as a cell model of HD. RL was observed in all cultures. The observed lasing thresholds were low—around 0.5–0.7 μJ/mm^2^ for all samples. The low pumping energy densities needed to obtain RL prevented us from damaging the cultures, even after extensive measurements.

We performed a multivariate statistical analysis of the 3000 RL spectra obtained. We randomly selected 70% of these spectra to build a model using two techniques: PCA and LDA. The other 30% remained untreated and were used to validate the model. As a result of this analysis, two discriminant functions were built. They were able to correctly classify the RL spectra used to build the model, with an average accuracy of 94.4% for the three types of cellular cultures.

To validate the model, we used two strategies: The first one, leave-one-out cross-validation, showed a rate of success of 94.1%. We also took the spectra that were not used in the model—30% of the original sample—and used the two discriminant functions to classify them. In this case, the model was able to assign each spectrum to its corresponding culture with an accuracy of 94.2%.

We are aware that these results were obtained in over-expressing cells, and further research on cells and tissues with endogenous expression of mHTT is needed in order to test this RL technique. We plan to conduct RL experiments to optically sense the brain and peripheral tissues of a genetic model of HD as a challenging follow-up of the present study.

## Figures and Tables

**Table 1:** Centroids of the discriminant functions for the three types of cell cultures.

Cell Type	*Discriminant Function*	
	LD1	LD2
NT	1.48(>0)	−1.52(<0)
HTT-Q23	1.03(>0)	1.72(>0)
HTT-Q74	−2.51(<0)	−0.20(≲0)

**Table 2:** Results of the classification and validation of the model.

			Predicted Cell Culture			
			NT	HTT-Q23	HTT-Q74	Accuracy
Training set	Original	NT	93.9	5.1	1.0	94.4%
		HTT-Q23	3.7	94.5	0.9	
		HTT-Q74	1.1	4.9	94.0	
	Cross-validated	NT	93.7	5.1	1.1	94.1%
		HTT-Q23	4.1	95.0	0.9	
		HTT-Q74	1.1	5.3	93.6	
Validation set		NT	95.0	4.7	0.3	94.2%
		HTT-Q23	3.7	95.3	1.0	
		HTT-Q74	1.3	6.3	92.3	

## Data Availability

The data presented in this study are available on request from the corresponding author. The data are not publicly available due to technical reasons.

## Abbreviations

The following abbreviations are used in this manuscript:

EtOH	Ethanol
FWHM	Full width at half maximum
HD	Huntington’s disease
LDA	Linear discriminant analysis
NT	Non-transfected
PCA	Principal component analysis
R6G	Rhodamine 6G
RL	Random lasing
